# Supplementary material for: A substrate-trapping strategy to find E3 ubiquitin ligase substrates identifies Parkin and TRIM28 targets
Source: Commun Biol. 2020 Oct 20;3:592. doi: 10.1038/s42003-020-01328-y (PMC7576197; doi:10.1038/s42003-020-01328-y)

Supplementary Figure 1: Substrate candidates can be efficiently identified by stably expressing the probe.

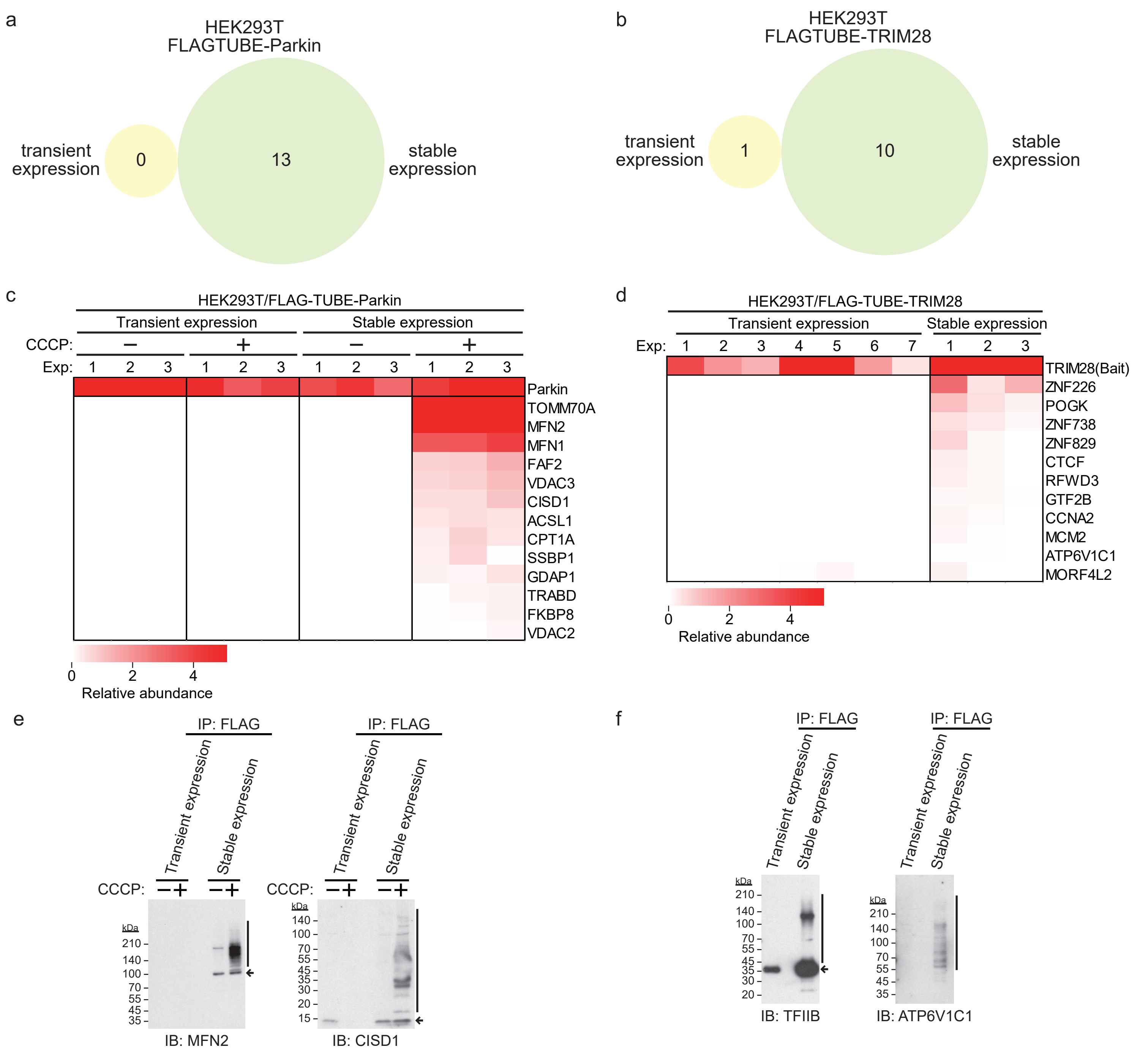

a, b Venn diagrams for identified substrate candidates in Parkin experiments with CCCP (10 µM) treatment for 1 h (a) and in TRIM28 experiments (b) in HEK293T cells. A probe in which TUBE was fused to the N terminus of each E3 ligase was used. **c, d** Substrate candidates for Parkin (c) and TRIM28 (d). Relative label-free quantification (LFQ) abundance is indicated by the color scale. Proteins with PSMs of >3 in at least one experiment (Exp) and >1 in at least two experiments were considered as substrate candidates for each E3 ligase. **e, f** Detection of ubiquitinated endogenous substrates. Cells expressing the FLAG-TUBE fused probe transiently or stably were harvested and anti-FLAG immunoprecipitates were analyzed by immunoblotting. Vertical bars and arrows denote the positions of ubiquitinated substrates and unmodified substrates, respectively.

Supplementary Figure 2: Overlapping of substrate candidates between HEK293T and HeLaS3 cells.

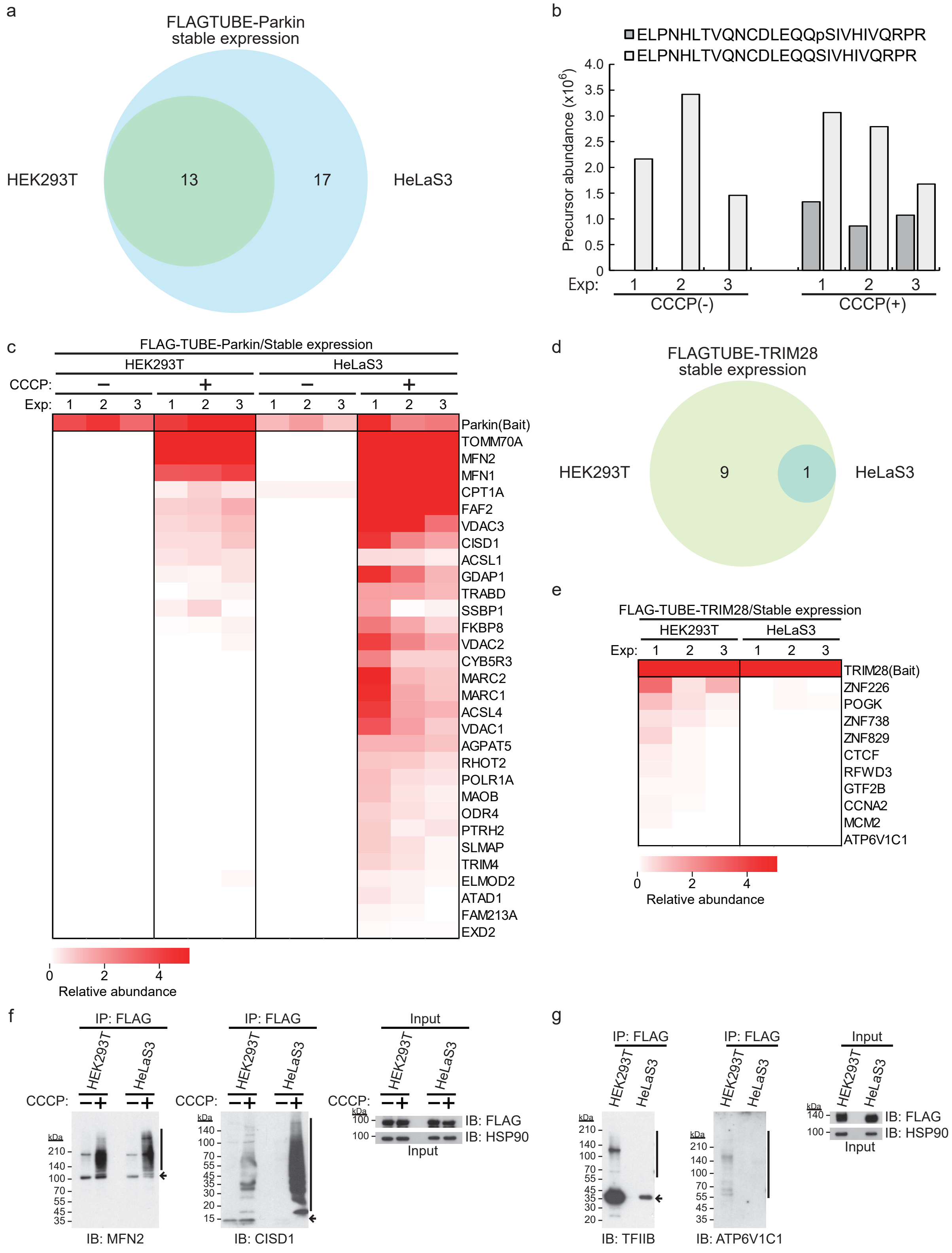

a, d Venn diagrams for identified substrate candidates in Parkin experiments with CCCP (10 µM) treatment for 1 h (a) and TRIM28 experiments (d) in HEK293T or HeLaS3 cells stably expressing each probe. Probes in which TUBE was fused to the N terminus of each E3 ligase were used. **b** Detection of Ser 65 phosphorylation of Parkin by MS. HeLaS3 cells expressing the FLAG-TUBE fused probe with (right) or without (left) CCCP (10 µM) treatment for 1 h were harvested and anti-FLAG immunoprecipitates were analyzed by MS. LFQ abundance of ELPNHLTVQNCDLEQQpSIVHIVQRPR or ELPNHLTVQNCDLEQQSIVHIVQRPR peptides of Parkin is shown. Three independent experiments were performed. **c, e** Substrate candidates for Parkin (c) and TRIM28 (e). Relative label-free quantification (LFQ) abundance is indicated by the color scale. Proteins with PSMs of >3 in at least one experiment (Exp) and >1 in at least two experiments were considered as substrate candidates for each E3 ligase. **f, g** Detection of ubiquitinated endogenous substrates. HEK293T or HeLaS3 cells expressing the FLAG-TUBE fused probe were harvested and anti-FLAG immunoprecipitates were analyzed by immunoblotting. Vertical bars and arrows denote the positions of ubiquitinated substrates and unmodified substrates, respectively.

Supplementary Figure 3: Validation of the substrate candidates for TRIM28 E3 ligases.

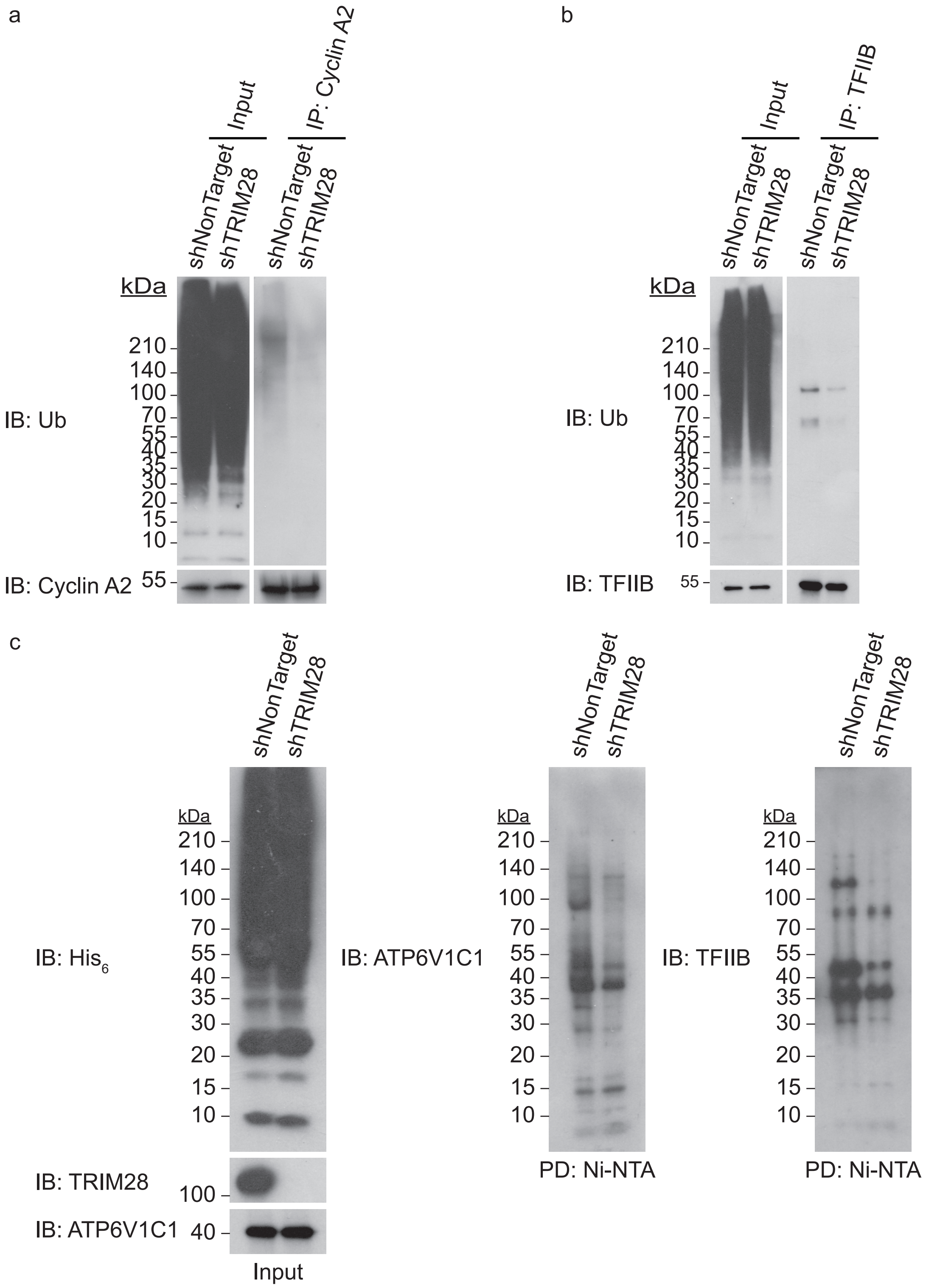

a, b HEK293T cells with stable knockdown of TRIM28 or corresponding control cells were harvested. Cell lysates were immunoprecipitated with an anti-Cyclin A2 antibody (a) or an anti-TFIIB antibody (b). The precipitates were analyzed by immunoblotting using an anti-Ub antibody. **c** pQCXIH-3×His6-ubiquitin, which encodes 3 copies of His6-tagged ubiquitin, was transfected into HEK293T cells with stable knockdown of TRIM28 or corresponding control cells. Forty-eight h after transfection, cells were harvested. Cell lysates were pulled down with Probond resin. The cell lysates (input) were immunoblotted with anti-His6, anti-TRIM28 and anti-ATP6V1C1 antibodies (left). The precipitates were analyzed by immunoblotting with anti-ATP6V1C1 (middle) and anti-TFIIB (right) antibodies.

Supplementary Figure 4: Validation of the substrate candidates for Parkin and TRIM28 E3 ligases.

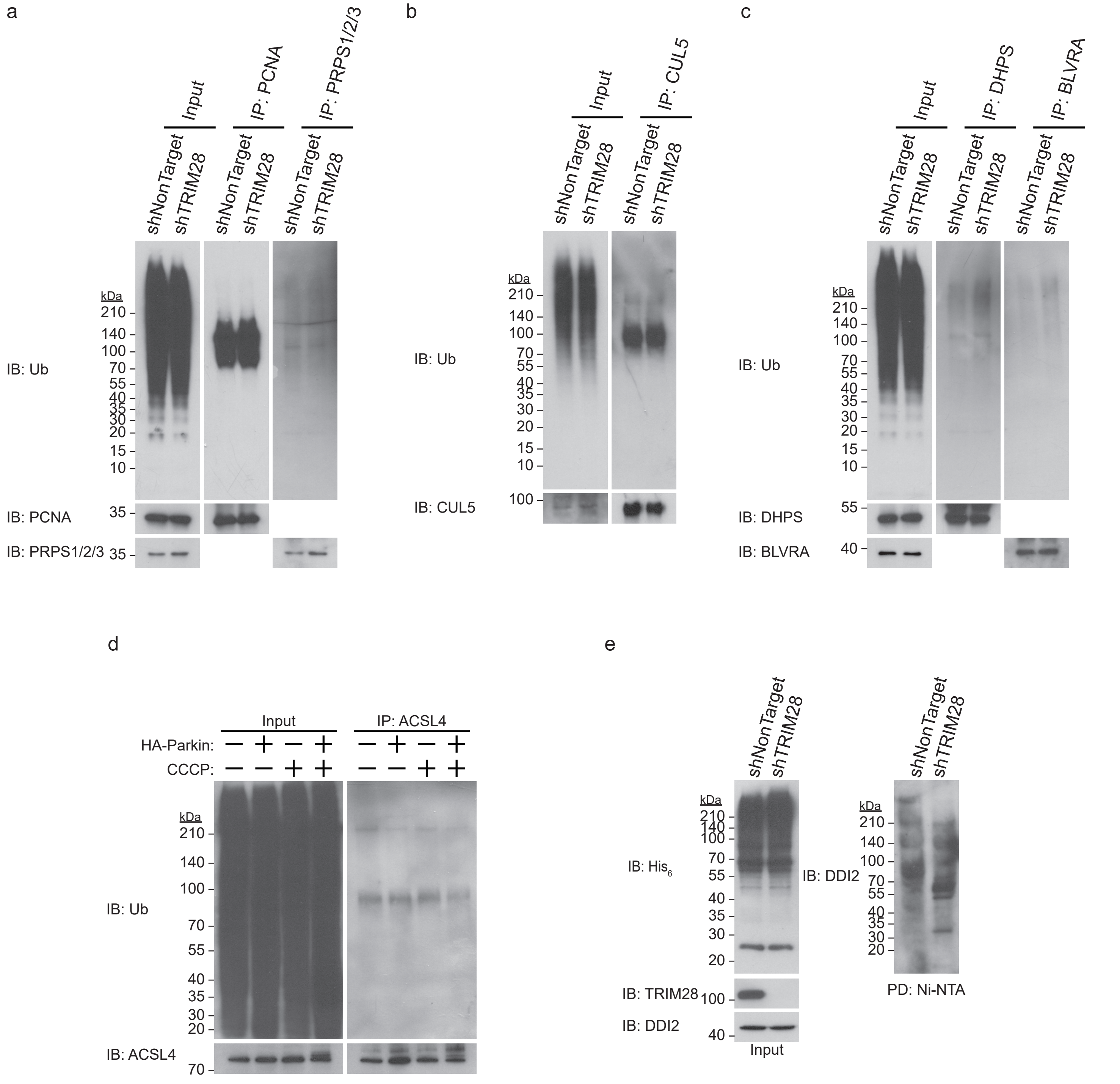

a-c HEK293T cells with stable knockdown of TRIM28 or corresponding control cells were harvested. Cell lysates were immunoprecipitated with an anti-PCNA antibody (a), an anti-PRPS1/2/3 antibody (a), an anti-CUL5 antibody (b), an anti-DHPS antibody (c), or an anti-BLVRA antibody (c). The precipitates were analyzed by immunoblotting with an anti-Ub antibody. **d** HEK293T cells stably expressing HA-Parkin or harboring an empty vector (Mock) were not treated with or were treated with CCCP (10 μM) for 1 h. Cell lysates were immunoprecipitated with an anti-ACSL4 antibody. The precipitates were analyzed by immunoblotting with an anti-Ub antibody. **e** pQCXIH-3×His6-ubiquitin was transfected into HEK293T cells with stable knockdown of TRIM28 or corresponding control cells. Forty-eight h after transfection, cells were harvested. Cell lysates were pulled down with Probond resin. The precipitates were analyzed by immunoblotting with an anti-DDI antibody.

Supplementary Figure 5: Landscape of the substrate candidates for Parkin and TRIM28 E3 ligases.

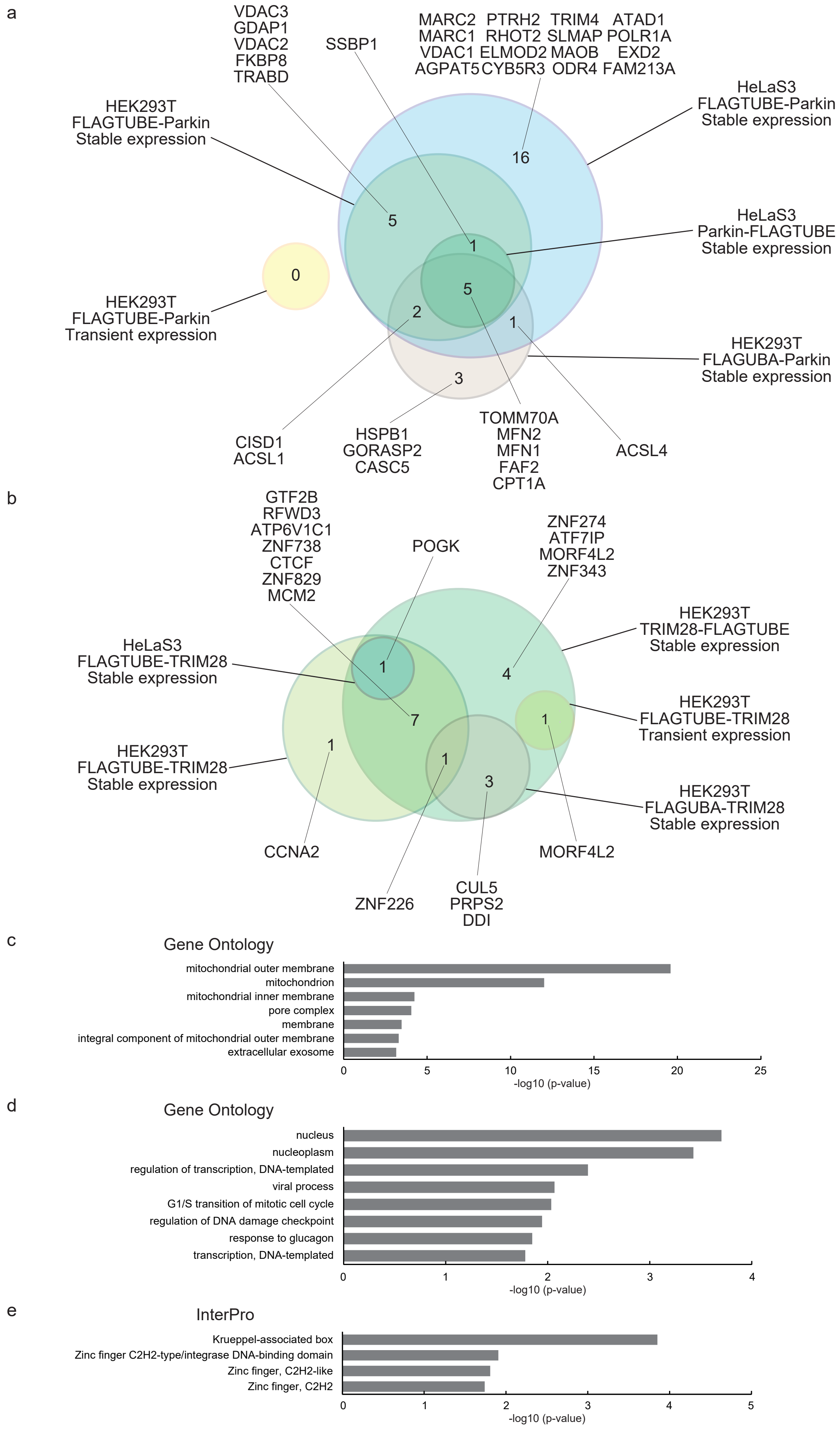

a, b Summary of identified substrate candidates in Parkin (a) and TRIM28 experiments (b). **c-e** Gene-annotation enrichment analysis for substrate candidates of Parkin (c) and TRIM28 (d, e). Gene Ontology cellular component (c), biological process (d) or InterPro (e) annotations enriched among the set of candidate proteins were identified using the DAVID Bioinformatics database. Graph shows the –log 10 (p-value) for each term.

Supplementary Figure 6: Full images of immuno blots

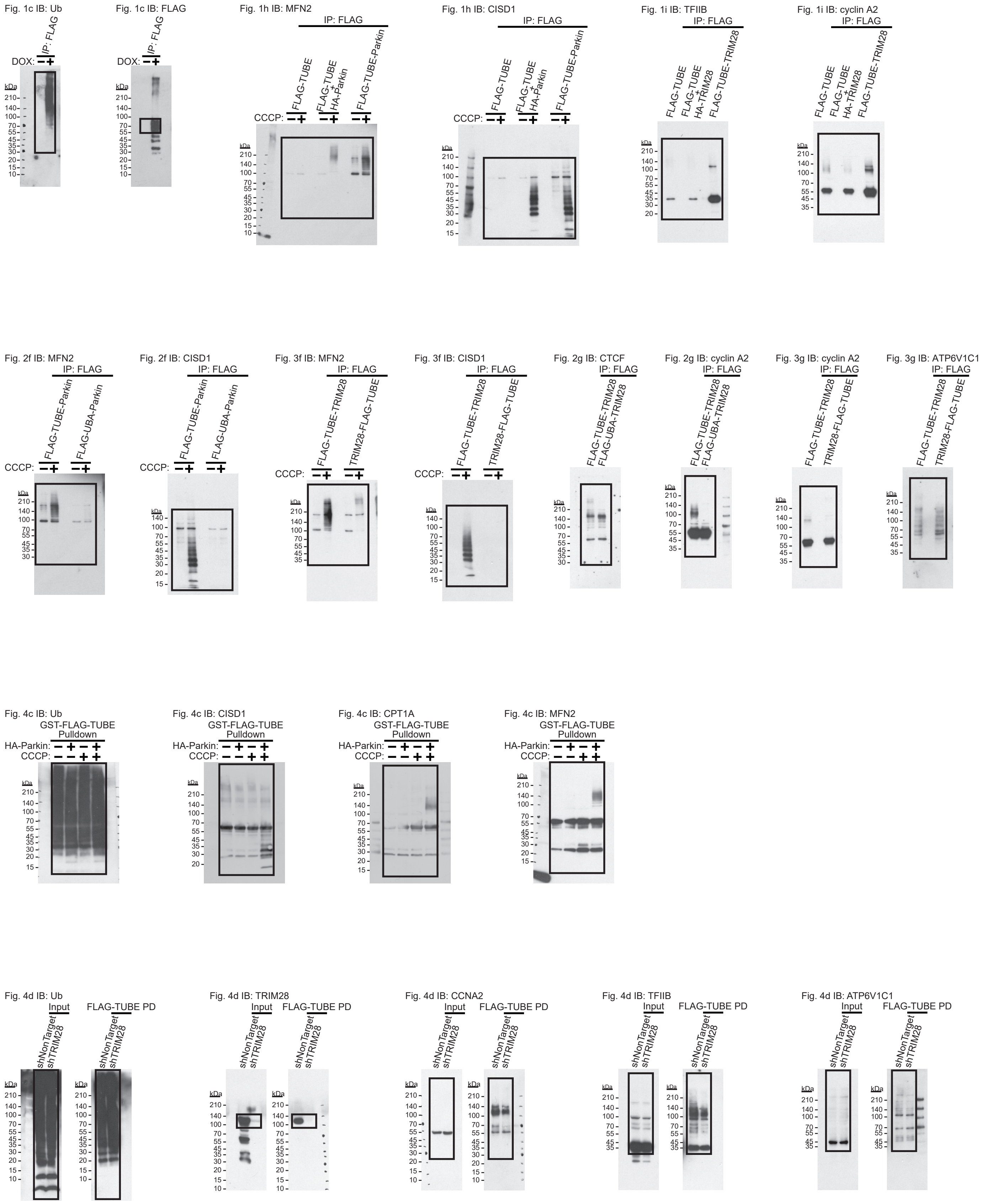

Fig. 5a

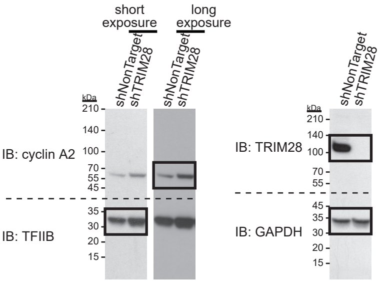

Fig. 5b

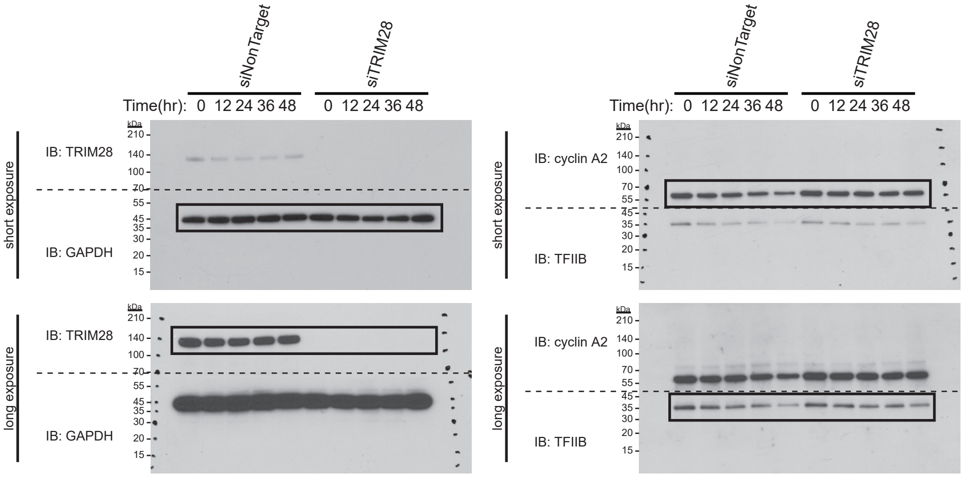

Fig. 5c

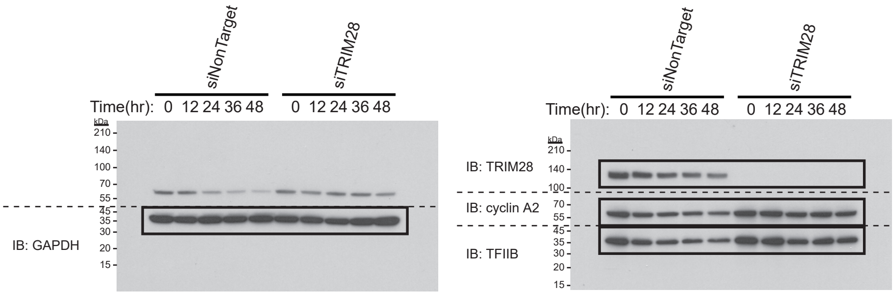

Fig. 5d

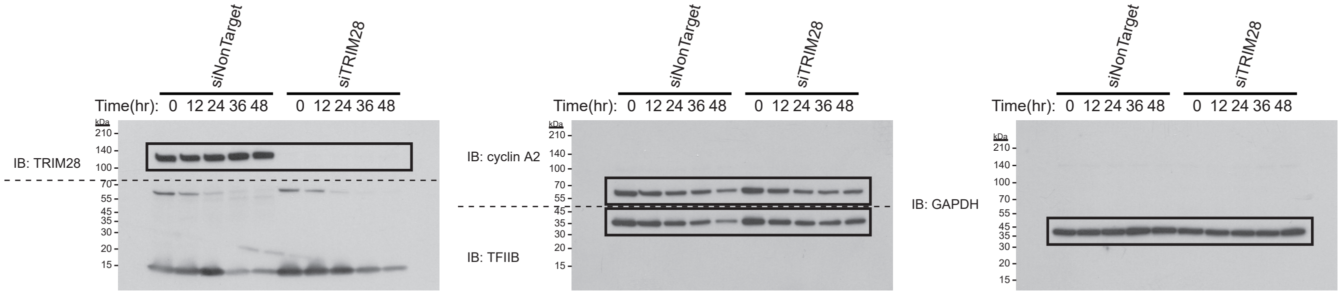

Fig. 5e

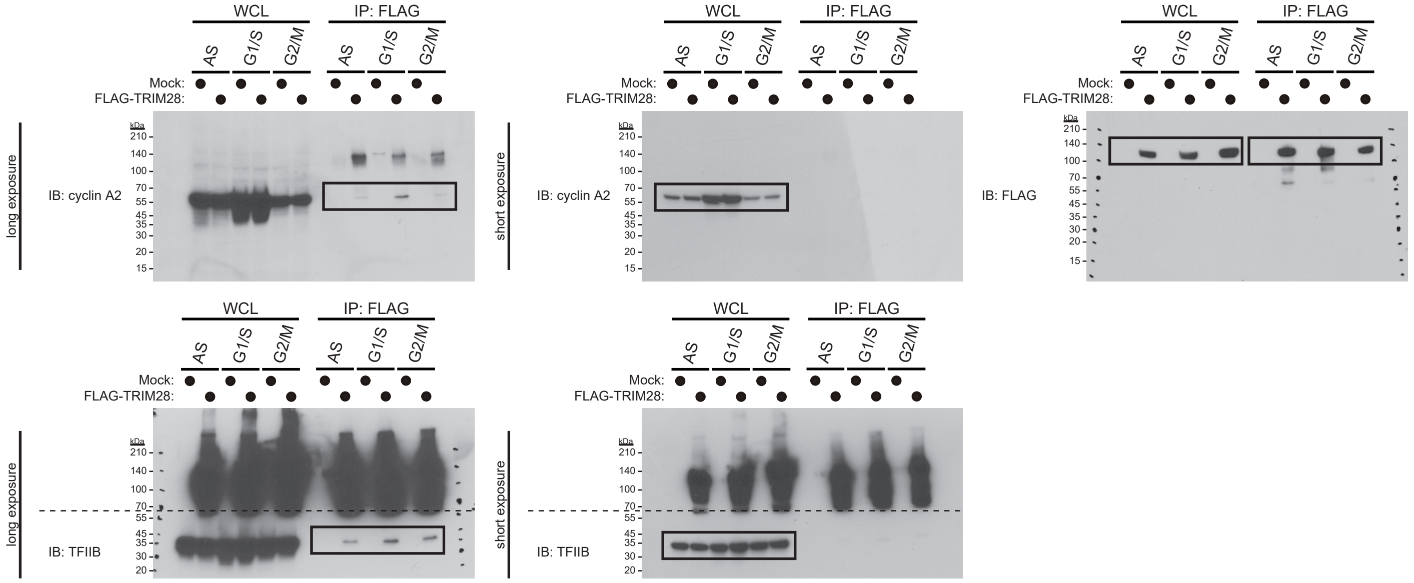

Supplementary Figure 1e
IB: MFN2

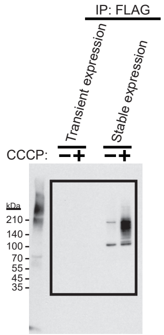

Supplementary Figure 1e
IB: CISD1

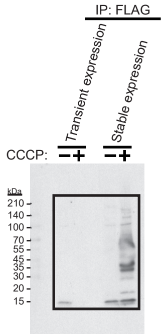

Supplementary Figure 1f
IB: TFIIB

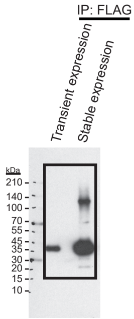

Supplementary Figure 1f
IB: ATP6V1C1

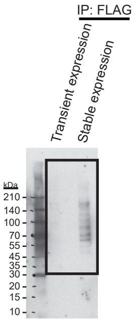

Supplementary Figure 2f
IB: MFN2

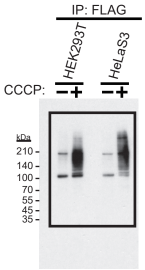

Supplementary Figure 2f
IB: CISD1

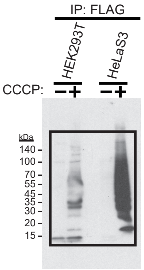

Supplementary Figure 2f
IB: FLAG

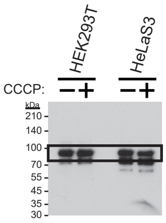

Supplementary Figure 2f
IB: HSP90

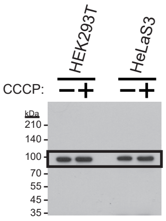

Supplementary Figure 2g
IB: TFIIB

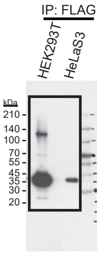

Supplementary Figure 2g
IB: ATP6V1C1

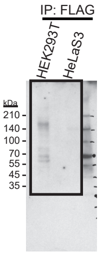

Supplementary Figure 2g
IB: FLAG

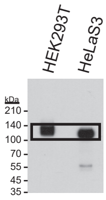

Supplementary Figure 2g
IB: HSP90

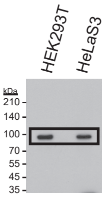

Supplementary Figure 3a
IB: Ub

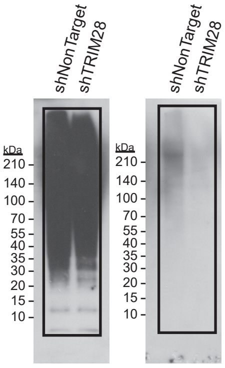

Supplementary Figure 3a
IB: cyclin A2

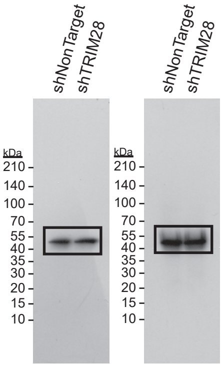

Supplementary Figure 3b
IB: Ub

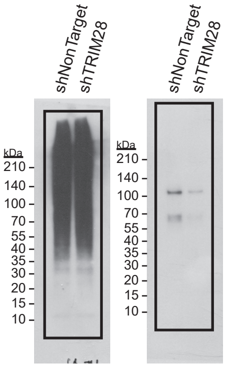

Supplementary Figure 3b
IB:TFIIB

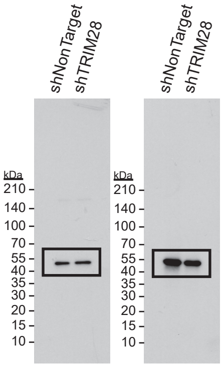

Supplementary Figure 3c
IB: His6

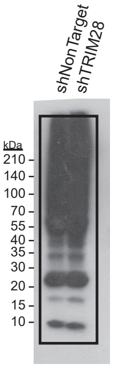

Supplementary Figure 3c
IB: TRIM28

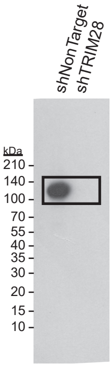

Supplementary Figure 3c
IB: TFIIB

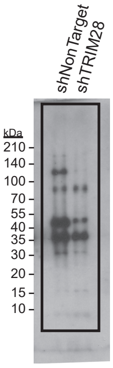

Supplementary Figure 3c
IB: ATP6V1C1

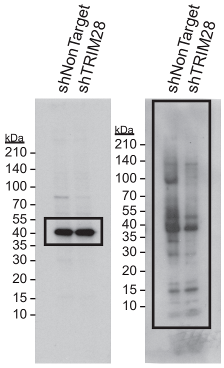

Supplementary Figure 4a
IB: Ub

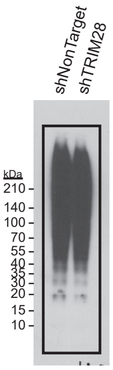

Supplementary Figure 4a
IB: Ub

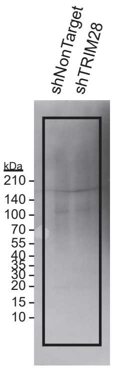

Supplementary Figure 4a
IB: PRPS1/2/3

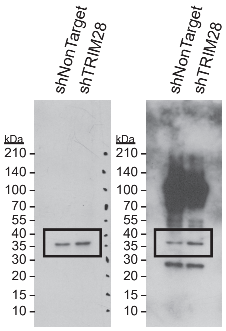

Supplementary Figure 4a
IB: Ub

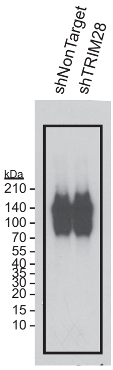

Supplementary Figure 4a
IB: PCNA

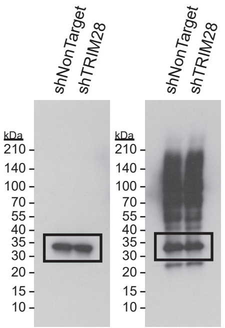

Supplementary Figure 4b
IB: Ub

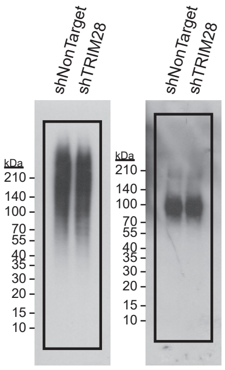

Supplementary Figure 4b
IB: CUL5

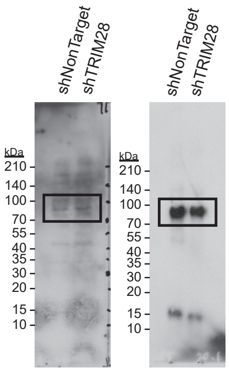

Supplementary Figure 4c
IB: Ub

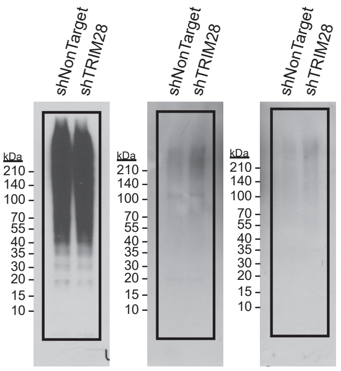

Supplementary Figure 4c
IB: DHPS

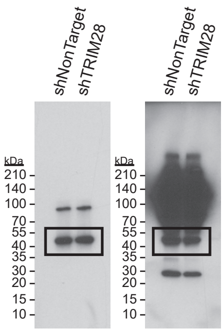

Supplementary Figure 4c
IB: BLVRA

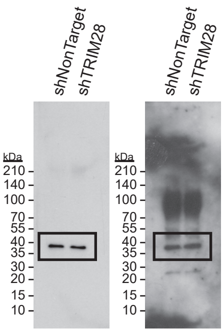

Supplementary Figure 4d
IB: Ub

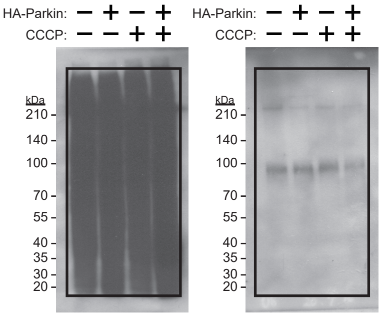

Supplementary Figure 4d
IB: ACSL4

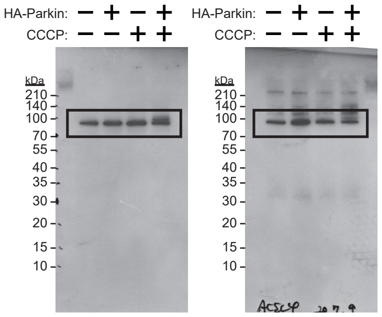

Supplementary Figure 4e
IB: His6

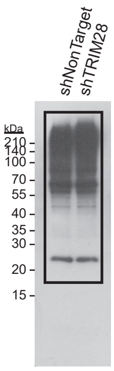

Supplementary Figure 4e
IB: TRIM28

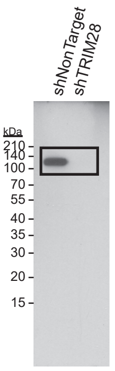

Supplementary Figure 4e
IB: DDI2

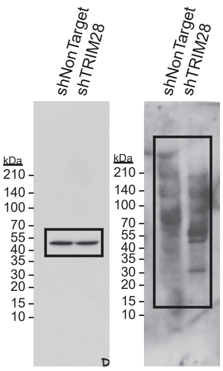

Supplement: Supplementary file 1 — Supplementary Information [file 42003_2020_1328_MOESM1_ESM.pdf]
