# Supplementary material for: A substrate-trapping strategy to find E3 ubiquitin ligase substrates identifies Parkin and TRIM28 targets
Source: Commun Biol. 2020 Oct 20;3:592. doi: 10.1038/s42003-020-01328-y (PMC7576197; doi:10.1038/s42003-020-01328-y)
Supplement: Supplementary file 3 — Description of Additional Supplementary Files [file 42003_2020_1328_MOESM3_ESM.pdf]

Description of Additional Supplementary Files

File Name: Supplementary Data 1
Description: **Non-specific ubiquitinated peptides (proteins) identified by the FLAG-TUBE probe not fused with E3 ligase, FLAG-TUBE-fused Parkin probe without stimulation, and probes fused with TRIM28 lacking its enzyme activity.** The ubiquitinated peptides identified by the FLAG-TUBE probe not fused with an E3 ligase or the FLAG-TUBE-fused Parkin probe, which is considered to be almost inactivated under unstimulated conditions, and ubiquitinated peptides identified with probes fused with E3 ligase with deletion of enzyme activity were indicated.

File Name: Supplementary Data 2
Description: **Ubiquitinated peptides and proteins identified by the Parkin probe.** Ubiquitinated peptides and proteins identified by all the Parkin probes examined in this study were listed. We compared samples with negative controls by label-free quantification (LFQ) abundance and by the total numbers of identified 3 sequences (PSMs) of ubiquitinated peptides. Proteins with PSMs of >3 in at least one experiment (Exp) and >1 in at least two experiments were considered as substrate candidates for each E3 ligase.

File Name: Supplementary Data 3
Description: **Ubiquitinated peptides and proteins identified by the TRIM28 probe.** Ubiquitinated peptides and proteins identified by all the TRIM28 probes examined in this study were listed. We compared samples with negative controls by label-free quantification (LFQ) abundance and by the total numbers of identified sequences (PSMs) of ubiquitinated peptides. Proteins with PSMs of >3 in at least one experiment (Exp) and >1 in at least two experiments were considered as substrate candidates for each E3 ligase.
